# Metabolic Connectivity and Hemodynamic-Metabolic Coherence of Human Prefrontal Cortex at Rest and Post Photobiomodulation Assessed by Dual-Channel Broadband NIRS

**DOI:** 10.3390/metabo12010042

**Published:** 2022-01-05

**Authors:** Xinlong Wang, Liang-Chieh Ma, Sadra Shahdadian, Anqi Wu, Nghi Cong Dung Truong, Hanli Liu

**Affiliations:** Department of Bioengineering, University of Texas at Arlington, 500 UTA Blvd, Arlington, TX 76019, USA; xinlong.wang@uta.edu (X.W.); mal4@vcu.edu (L.-C.M.); sadra.shahdadian@mavs.uta.edu (S.S.); anqi.wu@uta.edu (A.W.); nghicongdung.truong@mavs.uta.edu (N.C.D.T.)

**Keywords:** broadband near infrared spectroscopy, transcranial photobiomodulation, infraslow oscillation, prefrontal cortex, cytochrome c oxidase, resting state functional connectivity

## Abstract

Billions of neurons in the human brain form neural networks with oscillation rhythms. Infra-slow oscillation (ISO) presents three main physiological sources: endogenic, neurogenic, and myogenic vasomotions. Having an in vivo methodology for the absolute quantification of ISO from the human brain can facilitate the detection of brain abnormalities in cerebral hemodynamic and metabolic activities. In this study, we introduced a novel measurement-plus-analysis framework for the non-invasive quantification of prefrontal ISO by (1) taking dual-channel broadband near infrared spectroscopy (bbNIRS) measurements from 12 healthy humans during a 6-min rest and 4-min post transcranial photobiomodulation (tPBM) and (2) performing wavelet transform coherence (WTC) analysis on the measured time series data. The WTC indexes (IC, between 0 and 1) enabled the assessment of ipsilateral hemodynamic-metabolic coherence and bilateral functional connectivity in each ISO band of the human prefrontal cortex. At rest, bilateral hemodynamic connectivity was consistent across the three ISO bands (IC ≅ 0.66), while bilateral metabolic connectivity was relatively weaker. For post-tPBM/sham comparison, our analyses revealed three key findings: 8-min, right-forehead, 1064-nm tPBM (1) enhanced the amplitude of metabolic oscillation bilaterally, (2) promoted the bilateral metabolic connectivity of neurogenic rhythm, and (3) made the main effect on endothelial cells, causing alteration of hemodynamic-metabolic coherence on each side of the prefrontal cortex.

## 1. Introduction

The human brain accounts for 20% of the oxygen consumption and 25% of the glucose utilization in the human body, while it constitutes only 2% of the total body weight [1,2]. Cerebral energy metabolism is highly mediated by the vascular oxygen supply and neuronal/cellular oxidative respiration in the human brain [3,4]. Although the detailed mechanism of human cerebral metabolism is not fully understood, one major source was found to be the spontaneous oscillations originated from the blood vessel wall, called the vasomotion [5,6,7,8,9], which has an infra-slow oscillation (ISO) within 0.005–0.2 Hz [10,11]. In literature, cerebral metabolic activities with ISO are closely related to human cognitive functions [12]. Moreover, the malfunctioning of vasomotion has been associated with aging and diseases, such as atherosclerosis [13], cardiovascular disease [14], and Alzheimer’s Disease [15]. Therefore, it is beneficial to quantify cerebral metabolism in the ISO range for a better understanding of neurophysiology and potential causes of diseases.

Cerebral hemodynamic activity is known to be driven by the relaxation–contraction cycles of the vascular wall [16,17], which is independent from the heart beats and respiration [6,18]. Specifically, three main intrinsic/spontaneous ISO components of cerebral hemodynamic activity were resolved [19], namely, (1) endogenic oscillation (0.005–0.02 Hz) [20], (2) neurogenic oscillation (0.02–0.04 Hz) [21], and (3) myogenic oscillation (0.04–0.2 Hz) [22]. Briefly, the endogenic activity affects the vessel dilation–contraction cycles by releasing potent vasoactive factors, such as nitric oxide (NO), free radicals, prostacyclin, endothelium-derived hyperpolarizing factor, and endothelin [23,24]. Neurogenic activity modulates the vessel dilation–contraction cycles by the vasoactive ions and neurotransmitters from neurons/astrocytes [25]. Finally, myogenic activity mediates the vessel dilation–contraction cycles by relaxing and contracting the smooth muscle cells located on the vascular wall [22]. Common methods used to measure ISO include functional Magnetic Resonance Imaging (fMRI) [26], transcranial cerebral doppler (TCD) [27], and functional Near Infrared Spectroscopy (fNIRS) [12], followed by frequency-domain analysis [28]. However, these methods measure only hemodynamic vasomotion activities, not the activity of mitochondrial respiration. It is desirable to detect mitochondrial activity and its ISO because of its major role in cerebral metabolism and associated vasomotion [29].

Broadband near infrared spectroscopy (bbNIRS) is a non-invasive method that utilizes an abundant number of wavelengths to derive temporally fluctuated/oscillated concentration changes of oxygenated hemoglobin (Δ[HbO]), deoxygenated hemoglobin (Δ[HHb]) and oxidized cytochrome c oxidase (Δ[oxCCO]), the last enzyme in the mitochondrial respiration chain contributing in 95% of cellular/neuronal oxidative metabolism and ATP production [30,31,32]. Therefore, bbNIRS enables one to simultaneously record the oscillations of vascular oxygen supply in the blood stream and oxygen utilization by mitochondrial activities. The first success of the co-detection of ISOs of cerebral hemodynamic (i.e., Δ[HbO] and Δ[HHb]) and mitochondrial or metabolic (i.e., Δ[oxCCO]) activities and their interactions/interplays was achieved 20 years ago with bbNIRS taken non-invasively from the occipital region of normal participants at rest and under functional activations [33]. Similar ISO results were also observed in animal studies using camera-based invasive approaches to image cortical hemodynamic and metabolic activities [19,28].

Furthermore, our group employed bbNIRS to quantify changes of cerebral hemodynamic and metabolic activities of the human brain in response to transcranial photobiomodulation (tPBM) [34,35,36], a new form of non-invasive neuromodulation [37,38]. Specifically, tPBM delivers low-intensity light (through laser or light emitting diodes) to the human brain for the purpose of improving human cognition and/or treating a variety of brain diseases [37,38,39]. Our recent findings have provided quantitative support on the tPBM mechanism of action: tPBM enables the dose-dependent enhancement of Δ[HbO] and Δ[oxCCO] near the stimulation site on the human forehead [34,35,36] and increases functional connectivity across different cortical regions of the human brain [40]. However, our earlier bbNIRS/tPBM measurements focused on Δ[HbO] and Δ[oxCCO] alterations during tPBM with an interleaved protocol between the tPBM illumination and bbNIRS data acquisition. The sampling rate was 0.17 Hz, which prevented us from making ISO analysis at rest and post tPBM on the prefrontal cortex. Moreover, our earlier investigation was limited by a one-channel bbNIRS setup, giving no room to quantify the bilateral functional connectivity of the subject’s forehead either at rest or post tPBM.

Given the current research frontiers of ISO in cerebral hemodynamic and metabolic activities, algorithm development to determine resting state functional connectivity, as well as the bbNIRS technique capable of quantifying tPBM-enhanced metabolic activity, we had two objectives for this study. First, we introduced four key neurophysiological metrics to characterize the prefrontal cortex of human subjects at rest based on spontaneous ISO measured by dual-channel bbNIRS on bilateral sides of the participant’s forehead. These newly defined metrics included (1) bilateral hemodynamic connectivity, (2) bilateral metabolic connectivity, (3) interplay/coherence between cerebral hemodynamic-metabolic activities on the left and (4) right side of the prefrontal region. The second objective was to quantify and compare changes or alterations of these four metrics induced by tPBM. Specifically, after acquiring dual-channel bbNIRS with an improved sampling rate (~4 Hz), we utilized continuous wavelet transform (CWT) followed by wavelet transform coherence (WTC) analysis to establish respective indexes of coherence (IC) between respective pairs of Δ[HbO] and Δ[oxCCO] on either unilateral or bilateral sides across the three ISO frequency bands. The IC between two bilateral Δ[HbO] (or Δ[oxCCO]) would define and form bilateral functional cerebral hemodynamic (or metabolic) connectivity; the IC between the unilateral Δ[HbO] and Δ[oxCCO] oscillation is defined as cerebral hemodynamic-metabolic coherence on each prefrontal side of the human subjects. By the end, we would support our hypotheses: (1) dual-channel bbNIRS permits quantification of bilateral hemodynamic connectivity, bilateral metabolic connectivity, and unilateral coherence of cerebral hemodynamic-metabolic activities for a resting prefrontal cortex; (2) 8-min, right-forehead, 1064-nm tPBM facilitates significant augmentations of metabolic functional connectivity bilaterally and makes a key alteration of hemodynamic-metabolic coherence on each side of the prefrontal cortex.

## 2. Results

A total of 12 healthy humans (seven males and five females, mean ± SD age = 27.4 ± 6.1 years) participated in the experiments. Each subject had two separate visits, one for active tPBM and one for sham treatment. For both visits, we took dual-channel bbNIRS readings during the 6-min baseline period as resting-state measurements. Since no significant difference existed between these two sets of baseline parameters (from two visits), they were pooled as one grant group with a sample size of 24 for ISO analyses, followed by further quantifications of bilateral functional connectivity and cerebral hemodynamic-metabolic coherence based on WTC analysis.

### 2.1. Determination of ISO Coherence among Prefrontal Δ[HbO] and Δ[oxCCO] at Rest

#### 2.1.1. CWT Analysis for ISO Amplitude of Prefrontal Δ[HbO] and Δ[oxCCO] at Rest

**Figure 1:**
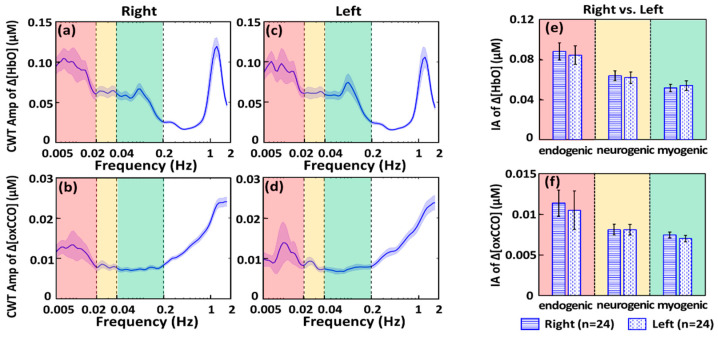
CWT-derived amplitude (Amp) spectra in resting state at the group level (*n* = 24) of (**a**) right prefrontal Δ[HbO]; (**b**) right prefrontal Δ[oxCCO]; (**c**) left prefrontal Δ[HbO]; and (**d**) left prefrontal Δ[oxCCO]. The blue-shaded band along each spectral curve marks the respective standard error of the mean. The red-, yellow-, and green-shaded boxes denote the endogenic, neurogenic, and myogenic frequency bands, respectively. Bilateral comparisons of spectrally averaged index of amplitude (IA) are shown for (**e**) Δ[HbO] and (**f**) Δ[oxCCO] from the resting prefrontal cortex of 24 bbNIRS measurements. Error bars indicate the standard error of the mean (*n* = 24).

After the 6-min-baseline time series of Δ[HbO] and Δ[oxCCO] were analyzed with CWT for both bbNIRS channels, we were able to obtain four time-frequency spectrograms for respective Δ[HbO] and Δ[oxCCO] on each lateral site. These spectrograms resulted in four amplitude spectra for Δ[HbO]_right_, Δ[HbO]_left_, Δ[oxCCO]_right_, and Δ[oxCCO]_left_ by temporally averaging the 6-min CWT amplitudes for each of the four metrics, as shown in Figure 1a–d. Figure 1a,c show that the CWT spectral amplitudes in the endogenic component (0.005–0.02 Hz) are dominant on both sides of the resting prefrontal cortex besides the narrow peak at 1.2 Hz caused by the cardiac activities. On the other hand, Figure 1b,d demonstrate the amplitude spectra of Δ[oxCCO] ISO of the resting prefrontal cortex. Symmetrical trends of ISO are obvious bilaterally, similar to the bilateral Δ[HbO] signals. However, it is noted that both amplitude spectra of Δ[oxCCO] exhibit stronger oscillation activities in the frequency range of 0.2–2 Hz without a cardiac peak at 1.2 Hz.

For frequency-band-specific comparison, for each subject, the spectral amplitude values in each amplitude spectrum were averaged, respectively, within endogenic, neurogenic, and myogenic frequencies, producing the Index of Amplitude (IA) for each of the three ISO bands. Cross-subject average of IA was further conducted to form group-level IAs, as shown in Figure 1e,f, to illustrate overall or global symmetrical trends of ISO of prefrontal Δ[HbO] and Δ[oxCCO] for a resting prefrontal cortex of normal humans. The fact that no significant difference existed between bilateral IAs of Δ[HbO] and Δ[oxCCO] implied uniform, consistent, and systemic distribution of the ISO amplitude in both cerebral hemodynamic and metabolic activities.

#### 2.1.2. WTC Analysis for ISO Coherence among Prefrontal Δ[HbO] and Δ[oxCCO] at Rest

**Figure 2:**
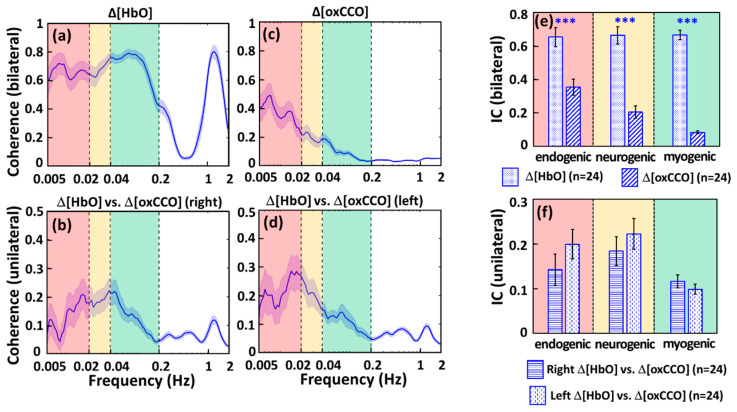
WTC-derived, resting-state coherence spectra at the group level (n = 24) are shown between (**a**) bilateral prefrontal Δ[HbO], (**b**) unilateral Δ[HbO] and Δ[oxCCO] on the right prefrontal side, (**c**) bilateral prefrontal Δ[oxCCO], and (**d**) unilateral Δ[HbO] and Δ[oxCCO] on the left prefrontal side. The blue-shaded band along with each spectrum marks the respective standard error of the mean. The red-, yellow-, and green-shaded boxes denote the endogenic, neurogenic, and myogenic frequency bands, respectively. Furthermore, spectrally averaged Indexes of Coherence (IC) are plotted at each ISO frequency band for (**e**) bilateral Δ[HbO] and bilateral Δ[oxCCO] and (**f**) unilateral interplay/coherence between Δ[HbO] and Δ[oxCCO] for each side of the prefrontal cortex at rest. Error bars indicate the standard error of the mean (*n* = 24). “***” indicates significant differences at *p* < 0.0001 based on paired *t*-tests.

After quantifications of the four amplitude spectra of Δ[oxCCO] and Δ[HbO] ISOs of the resting prefrontal cortex, WTC analysis resulted in four sets of coherence spectra between (a) bilateral prefrontal Δ[HbO], (b) unilateral Δ[HbO] versus Δ[oxCCO] on the right prefrontal side, (c) bilateral prefrontal Δ[oxCCO], and (d) unilateral Δ[HbO] versus Δ[oxCCO] on the left prefrontal side, as shown in Figure 2a–d. Strong coherences between bilateral Δ[HbO]s existed through the three ISO frequency bands (0.005–0.2 Hz) besides the cardiac frequency (i.e., 1.2 Hz) (see Figure 2a), while dominant coherences between bilateral Δ[oxCCO]s occurred at endogenic frequency (see Figure 2c).

This set of observations implied that the bilateral cerebral hemodynamic connectivity of the prefrontal cortex at rest was rather strong across the entire ISO range, while the bilateral metabolic connectivity was strongest in the endogenic frequency as compared to the neurogenic and myogenic frequency bands, but still much weaker than its counterpart of the cerebral hemodynamic connectivity. Furthermore, unlike the case in Δ[HbO], the bilateral coherence of Δ[oxCCO] at 1.2 Hz appears to be negligible, supporting the claim/expectation that Δ[oxCCO] obtained in our method would be contaminated minimally from the crosstalk/residual with Δ[HbO]. The distinct spectral shapes and strengths seen in Figure 2a,c as well as the absence of cardiac coherence at 1.2 Hz in Figure 2c verify the independency between the temporal association of Δ[oxCCO] and Δ[HbO].

Furthermore, as an indicator of cerebral hemodynamic-metabolic coherence, the unilateral coherence spectra between Δ[HbO] and Δ[oxCCO] on both prefrontal sides exhibited similarity in both amplitude and spectral trends (Figure 2b,d); specifically, they both had relatively smaller coherence at the cardiac frequency of 1.2 Hz compared to that in the ISO range. This set of observations indicated that the major interplay or coherence between Δ[HbO] and Δ[oxCCO] occurred or was associated in the ISO frequencies, rather than caused by the systemic heart beats.

In addition, for frequency-band-specific comparison, we introduced or defined a spectrally averaged index of coherence (IC) for each of the three ISO bands in the four coherence spectra (Figure 2a–d). Respective ICs are plotted in Figure 2e,f, presenting several notable features: (a) Relatively stable bilateral hemodynamic connectivity was evident for the prefrontal cortex at rest across the entire ISO region with a global IC constant of 0.66. (b) Overall, bilateral ICs for metabolic connectivity were relatively weaker than those for hemodynamic connectivity, particularly in the neurogenic and myogenic frequency bands. (c) No significant difference existed between Δ[HbO] and Δ[oxCCO] coherences on each side of the prefrontal cortex, confirming bilateral resemblance of cerebral hemodynamic-metabolic coherence in the prefrontal cortex at rest.

### 2.2. tPBM-Induced Alterations in ISO Coherence among Prefrontal Δ[HbO] and Δ[oxCCO]

#### 2.2.1. tPBM-Induced Changes in IA of ISO of Prefrontal Δ[HbO] and Δ[oxCCO]

**Figure 3:**
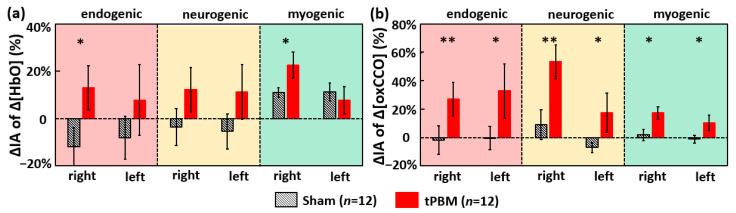
Baseline-normalized percentage changes of IA (i.e., ΔIA) at the bilateral prefrontal cortex for (**a**) Δ[HbO] and (**b**) Δ[oxCCO] across three ISO frequency bands. tPBM- and sham-induced ΔIA values are marked by red and black bars, respectively (*n* = 12). Error bars indicate the standard error of the mean in each case. The red-, yellow- and green-shaded boxes denote the endogenic, neurogenic, and myogenic bands, respectively. “*” indicates *p* < 0.05, and “**” indicates *p* < 0.01 based on paired *t*-tests between tPBM and sham experiments.

To determine tPBM-induced effects in cerebral hemodynamic-metabolic coherence and functional connectivity, we had to compute the tPBM/sham-induced changes in oscillation amplitude, namely, changes in IA (ΔIA) between 6 min pre tPBM and 4 min post tPBM. Thus, besides the IA values obtained in Section 2.1 during the 6-min baseline (i.e., pre-tPBM/sham) period, the IA values across the three ISO bands during the 4-min post-tPBM/sham period were computed for each of the four cerebral hemodynamic and metabolic metrics, followed by respective baseline normalization resulting in a percentage change (See Section 4.4 for details). Figure 3a shows that, compared to sham, tPBM significantly enhanced ΔIA of Δ[HbO] by 13% and 23% in the endogenic and myogenic bands, respectively, in the right prefrontal cortex. Furthermore, Figure 3b illustrates that tPBM increased ISO amplitudes of Δ[oxCCO] significantly in all frequency bands, particularly in both endogenic and neurogenic bands, on both sides of the prefrontal cortex.

#### 2.2.2. tPBM-Induced Changes in IC of ISO among Prefrontal Δ[HbO] and Δ[oxCCO]

**Figure 4:**
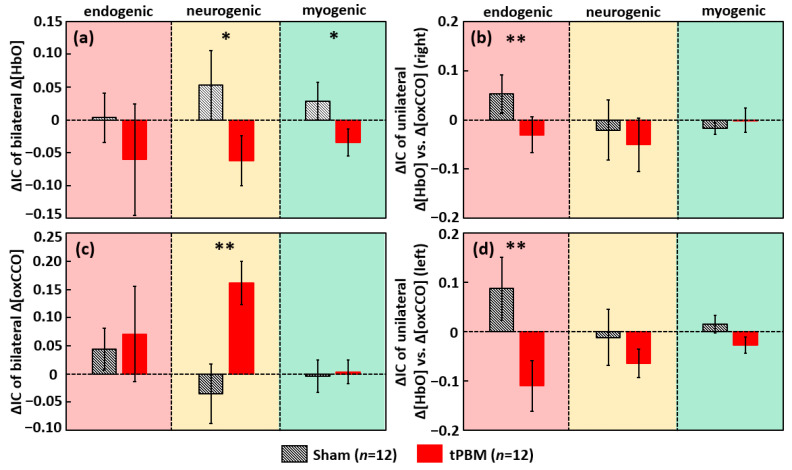
Baseline-subtracted changes of IC, ΔIC, for (**a**) bilateral Δ[HbO] coherence, (**b**) unilateral Δ[HbO] vs. Δ[oxCCO] coherence (right), (**c**) bilateral Δ[oxCCO] coherence, and (**d**) unilateral Δ[HbO] vs. Δ[oxCCO] coherence (left). tPBM- and sham-induced ΔIC values are marked by red and black bars, respectively (*n* = 12). Error bars indicate the standard error of the mean in each case. The red, yellow, and green-shaded boxes denote the endogenic, neurogenic, and myogenic bands, respectively. “*” indicates *p* < 0.05, and “**” indicates *p* < 0.01 based on paired *t*-tests between tPBM and sham experiments.

To examine the tPBM/sham-induced alterations in cerebral hemodynamic-metabolic coherence and bilateral functional connectivity, we again utilized WTC and computed the newly defined IC values during the 4-min recovery period for bilateral Δ[HbO], bilateral Δ[oxCCO], and unilateral Δ[HbO] vs. Δ[oxCCO] on each lateral side. Then, changes in ICs (ΔIC) between post and pre stimulation were obtained for separate tPBM and sham conditions for each of the four respective coherence cases, as summarized in Figure 4. Accordingly, we observed that, as compared to sham, (1) tPBM reduced coherence or desynchronized the bilateral prefrontal Δ[HbO] in neurogenic and myogenic bands significantly (Figure 4a); (2) tPBM also desynchronized the interplay between Δ[HbO] and Δ[oxCCO] in the endogenic band on each side of the prefrontal cortex (Figure 4b,d); and (3) tPBM significantly enhanced the synchronization of the bilateral Δ[oxCCO] coherence in the neurogenic band in the prefrontal cortex (Figure 4c).

## 3. Discussion

In this study, we implemented a two-channel bbNIRS system and objectively measured time-dependent cerebral hemodynamic and metabolic activities with ISO of the bilateral prefrontal cortex of healthy participants under resting and tPBM/sham conditions. The results indicate several significant findings and some limitations.

### 3.1. Feasibility of bbNIRS in Monitoring Spectral Amplitudes of Human Cerebral ISO at Rest

The infraslow oscillation (0.005–0.2 Hz) originated from the intrinsic vasomotion is believed to be a dominant and systemic activity that is highly associated to the cerebral neurophysiology of endogenic (0.005–0.02 Hz), neurogenic (0.02–0.04 Hz), and/or myogenic (0.04–0.2 Hz) functions in the healthy human brain [20,21,22,41]. Moreover, diminished or impaired cerebral hemodynamic and metabolic infraslow activities (CMIA) have been reported as an indicator to numerous neurological or metabolic diseases, such as cardiovascular disease, Alzheimer’s Disease, hypertension and stroke [14,15,24]. Thus, it is highly desirable to develop a non-invasive methodology and portable device to quantify/monitor CMIA with high sensitivity for the early detection of respective diseases and a better understanding of neurophysiology in the healthy human brain. In this study, we introduced the spectral oscillation amplitude and coherence of blood oxygenation (i.e., Δ[HbO]) and oxidized cytochrome c oxidase (i.e., Δ[oxCCO]) non-invasively measured by bbNIRS from the human forehead, demonstrating the high feasibility of bbNIRS to serve as a new monitoring device for quantifying CMIA of in the human brain at rest.

Figure 1 illustrated the bilateral ISO spectra of Δ[HbO] and Δ[oxCCO] between 0.005 and 0.2 Hz; the observed spectral shapes are well consistent with previous reports given by other groups using the same or different detection methods/techniques [16,17,33,42,43]. The advantage of this current study over others is the concurrent quantification of bilateral Δ[HbO] and Δ[oxCCO] time series, allowing for analysis and observation on the symmetry of ISO spectra of the bilateral prefrontal cortex. All the results in Figure 1 demonstrate significantly symmetrical features in spectral shapes and IAs of Δ[HbO] and Δ[oxCCO] between two lateral prefrontal regions across all three ISO ranges in the rest human brain. Furthermore, weaker oscillation in 0.2–1 Hz was observed for Δ[HbO] at the bilateral prefrontal cortex. This observation was also consistent with previous reports on hemodynamic activities in CMIA [9,33,44].

### 3.2. Feasibility of bbNIRS to Detect Human ISO Coherence of Prefrontal Cortex at Rest

The coherence between bilateral Δ[HbO]s reflects intrinsic hemodynamic connectivity, while the coherence between bilateral Δ[oxCCO]s reflects intrinsic metabolic networks [33]. Moreover, unilateral coherence between Δ[HbO] and Δ[oxCCO] on each side of the forehead can signify cerebral hemodynamic and metabolic coherence. Wang et al. [32,34] reported that this interplay/relationship was photobiomodulated by this 1064 nm laser.

In this study, we illustrated strong and constant bilateral coherence of cerebral hemodynamic (i.e., Δ[HbO]) activities in the human prefrontal cortex at rest with an average IC value of 0.66 across the three ISO ranges between 0.005 and 0.2 Hz (see Figure 2a,e). This observation implied that the major hemodynamic connectivity between bilateral prefrontal regions involves slow oscillational activities of endothelial cells (endogenic band, 0.005–0.02 Hz), inter-neurons (neurogenic band, 0.02–0.04 Hz), and smooth vascular muscle cells (myogenic band, 0.04–0.2 Hz) [12,45,46]. Prefrontal connectivity is of essential significance in reflecting cognitive functions and the severity of cognition-impairing diseases [47,48,49,50,51]. Thus, various reports have been published to reveal Δ[HbO]-derived hemodynamic connections within the prefrontal cortex [48,52,53,54,55].

Our findings on the high bilateral coherence of prefrontal hemodynamics (IC = 0.66) of the resting human brain with ISO are highly consistent with those observed by others. Cordes et al. reported that fMRI-derived functional connectivity is mainly contributed by the slow frequency oscillation (0–0.1 Hz) in the human brains [56]. Sasai et al. [57] in 2014 implemented concurrent NIRS and fMRI to monitor cerebral hemodynamics of the human brain at rest. There are two major consistent findings between ours and theirs. First, they reported that 0.01–0.03 Hz and 0.07–0.09 Hz mainly contributed to resting state functional connectivity. Very consistently, these two frequency bands fall into or match well the spectral range of ISO where we showed strong bilateral coherence or connectivity, as seen in Figure 2a,e. Second, Sasai et al. reported an average of ~0.6 cerebral coherence derived from their NIRS (Δ[HbO]) and ~0.4–0.5 from fMRI (BOLD) signals across different brain regions, indicating the high connectivity of the human brain. Their coherence values (particularly derived from a two-wavelength NIRS) and ours for Δ[HbO] are in excellent agreement.

In the meantime, the IC values for Δ[oxCCO] exhibited that cerebral metabolic connectivity between bilateral prefrontal regions involves mainly endothelial cells (0.005–0.02 Hz) in ISO activities as compared to the ICs in the other two ISO frequency bands (see Figure 2c,e). This is one of the novel findings of this study, revealing the feasibility to quantify prefrontal metabolic connectivity (by IC at endogenic frequency) in the human prefrontal cortex at rest. Such a quantifiable metric may serve as a biomarker/feature to characterize the metabolic activity of the human prefrontal cortex at rest.

Strong coherence at 1.2 Hz seen in Figure 2a between the bilateral Δ[HbO] time series is expected due to the cardiac and systemic signal. However, minimal coherence was detected in Figure 2c at this frequency between bilateral Δ[oxCCO] time series, indicating the reduced driving source from the heartbeat to Δ[oxCCO] oscillation. Although a number of computational and experimental studies have demonstrated that the Δ[oxCCO] quantified from bbNIRS is not significantly affected by the crosstalk from Δ[HbO], it has been questioned for years whether the calculated Δ[oxCCO] was originated from the crosstalk or residual of Δ[HbO] [58,59,60,61]. The absence of coherence between bilateral Δ[oxCCO] oscillations at 1.2 Hz (Figure 2c) confirmed the minimum crosstalk between calculated Δ[HbO] and Δ[oxCCO] by the multi-linear regression algorithm of bbNIRS [32,34].

Moreover, we observed similar spectral shapes of unilateral coherence between Δ[HbO] and Δ[oxCCO] at the same site of the right and left forehead, respectively (Figure 2b,d). This set of results indicate symmetrical cerebral hemodynamic-metabolic connection or coherence between the bilateral prefrontal cortex of the healthy and rest human brain, with further confirmation of no significant difference between bilateral ICs shown in Figure 2f. Furthermore, the observation that the major Δ[HbO] vs. Δ[oxCCO] coherence occurred in the entire ISO range of 0.005–0.2 Hz indicated a close cerebral hemodynamic-metabolic interaction originated from all endogenic, neurogenic, and myogenic functions. The spectral feature of Δ[HbO] vs. Δ[oxCCO] coherence was consistent with previous studies from other groups [33]. In addition, a small/negligible coherence peak at ~1.2 Hz seen in both coherence spectra (Figure 2b,d) indicated that cerebral hemodynamic-metabolic coherence was calculated free of crosstalk from the cardiac oscillation or fluctuation. The minimal coherence between unilateral Δ[HbO] and Δ[oxCCO] oscillations at 1.2 Hz (Figure 2b,d) verified again that the quantified Δ[oxCCO] based on bbNIRS measurements did not result from the crosstalk or residuals of Δ[HbO].

### 3.3. bbNIRS as a Promising Non-Invasive Tool for Absolute Value Detections

Because of its non-invasiveness, portability, low-cost, easiness of use, and potential of measuring metabolic activities [59,62,63], NIRS-based techniques are clinically preferred as a bedside monitoring tool. However, the NIRS technique has not been adopted by mainstream medicine; one reason for this may be its relative measures. For example, the diffuse correlation spectroscopy only detects a relative blood flow index [64], and functional NIRS detects only relative concentration changes [65]. This hindered the NIRS-based technique from being used in resting-state-based measurements for disease diagnosis. It is impossible to conduct task-based measurements for patients with dementia or other mental/physical disabilities. Although the frequency-domain- and time-domain-based NIRS systems can produce absolute values of hemodynamic parameters as outcomes, they require high-cost instrumentation [66,67]. In this study, we demonstrated a non-invasive, dual-channel bbNIRS system with wavelet-based analysis that enabled us to introduce several key neurophysiological metrics (with absolute ICs) to characterize bilateral functional connectivity and unilateral cerebral hemodynamic-metabolic coherence on the prefrontal cortex of human subjects at rest. Since the newly developed connectivity and coherence metrics are quantified or represented by coherence (i.e., IC values) ranging between 0 and 1, they can be acquired at resting state as an absolute quantity. Thus, they may be able to serve as promising biomarkers to diagnose/detect cerebral abnormalities in cerebral hemodynamic and/or metabolic activities by simple and short resting-state recordings. In summary, the outcomes in Figure 1 and Figure 2 produced by dual-channel bbNIRS were very promising to motivate us for further clinical research and for sooner translation of bbNIRS to clinical uses.

### 3.4. tPBM-Enhanced Spectral Amplitude of Ipsilateral Δ[HbO] and Bilateral Δ[oxCCO] ISO

The observation of significant increases in Δ[HbO] oscillation amplitude, ΔIA, in endogenic and myogenic bands (13% and 23%) on the right prefrontal cortex post tPBM implies that it enables one to locally/ipsilaterally increase the hemodynamic activities originated from the endothelial and smooth muscle oscillations within 4 min (or longer) post tPBM. This set of results are in good agreement with our previous findings [34]. The dual-channel bilateral bbNIRS measurement with frequency-domain analysis in this study enabled us to reveal tPBM-enhanced hemodynamic responses on bilateral sides and tPBM-induced alteration in ISO in their respective physiological sources.

The basic mechanism of tPBM was believed as the photo-oxidation of CCO in mitochondria [68,69,70]. As seen in our results (Figure 3b), right-forehead tPBM enabled Δ[oxCCO] spectral magnitude, ΔIA, in both ipsilateral and contralateral enhancement in all three ISO bands. This implied that, as the sources of ISO [17,20,21,22], the endothelial cells, inter-neurons, and vascular smooth muscle cells were all activated by tPBM. Since all three types of cell/neurons contain a large amount of mitochondria/CCO [71,72], the exposure of them to 1064-nm light would photobiomodulate their mitochondrial CCO and activate their respective ISO oscillations. It was the first time to have observed tPBM being able to promote spectral amplitude of Δ[oxCCO] on bilateral sides, hinting the possibility of tPBM-enhanced bilateral Δ[oxCCO] connectivity within ISO frequencies.

### 3.5. tPBM-Induced Alteration in Bilateral Functional Connectivity and Unilateral Coherence in ISO of [HbO] and [oxCCO]

The observation (Figure 4a) of decreased coherence of bilateral Δ[HbO] in neurogenic and myogenic bands indicated that the original strong bilateral hemodynamic coherence during baseline was perturbed by right frontal tPBM photo-oxygenation. This may be explained as follows: the tPBM-induced significant increment in concentration and oscillation amplitude of Δ[HbO] on the right forehead caused the imbalanced increasing/alteration rate of hemodynamic fluctuations on the right prefrontal side with respect to the left side, leading to the reduction in bilateral synchronization of Δ[HbO]. On the other hand, the results form Figure 4c reveal a strong and significant rise in bilateral coherence of Δ[oxCCO] in the neurogenic band. The potential explanation is that the dendrites of neurons possess a large number of mitochondria (CCO), facilitating tPBM effects on neuronal activations [73]. Neurogenic functions are mediated by neuronal activities, whose activation can be transmitted/propagated distally to the other lateral side of the brain [74,75,76]. In the case of right prefrontal tPBM, accordingly, local neuron activities would increase on the ipsilateral side and in the meantime trigger the neuronal activations on the contralateral side. Thus, bilateral neurogenic synchronization of Δ[oxCCO] was enhanced, leading to an enhanced coherence of neuro-network connectivity [77].

Furthermore, the decreased coherence between Δ[HbO] and Δ[oxCCO] on each unilateral side (Figure 4b,d) at the endogenic band means a reduction in coherence/interplay between Δ[HbO] and Δ[oxCCO] at the same location. In healthy neurons/cells, CCO consumes oxygen transported from HbO and produces ATP for cellular/neuronal energy [71,72]. The decrease/desynchronization of Δ[HbO] vs. Δ[oxCCO] coherence might be interpreted below. We showed in our previous studies that tPBM promoted both concentrations of Δ[oxCCO] and Δ[HbO] linearly in amplitude [34,36], without knowing their coherent or coherence relationship. It is also known that tPBM would release nitric oxide (NO) which stimulates vessel dilation [78]. Accordingly, we speculated two sources contributing to our measured Δ[HbO] by bbNIRS: one was a Δ[oxCCO]-driven component [34] and the other a vessel-dilation-driven component. With this speculation, it is understandable or expected that Δ[HbO] and Δ[oxCCO] would be less coherent in their ISO when Δ[HbO] oscillation was also driven by NO-related contribution besides Δ[oxCCO] fluctuation. The fact that only the endogenic rhythm of Δ[HbO] vs. Δ[oxCCO] coherence was lowered (Figure 4b,d) suggests that the main effect on cerebral hemodynamic-metabolic coherence by tPBM occurs only on endothelial cells [23,79]. This speculative finding seems to match well the NO effect on endothelial cells on blood vessels.

In theory, neurovascular coupling refers to the mechanism linking the neural activity to the cerebral hemodynamic activity. Since mitochondria are within neurons, dynamic changes in [oxCCO] measured by bbNIRS reflects neural or neuronal activity. The unilateral coherence between mitochondrial metabolism and vascular hemodynamics quantified in our study should closely associate or correlate with neurovascular coupling, at least indirectly. Thus, the ability of tPBM being able to modulate [HbO] vs. [oxCCO] coherence implies that tPBM may be able to facilitate the non-invasive modulation of neurovascular coupling. We expect that concurrent measurements of electroencephalogram (EEG) and bbNIRS during and post tPBM in the human brain will enable us to demonstrate tPBM-induced changes of neurovascular coupling. This expectation will be examined and confirmed in our future studies.

In summary, the results shown in Figure 4 demonstrate significant tPBM-evoked enhancement on bilateral metabolic connectivity or neurogenic synchronization of Δ[oxCCO] and alterations of ipsilateral cerebral hemodynamic-metabolic coherence in the prefrontal cortex. These sets of results confirm the promising perspective of tPBM in treating different neurological and cerebral vascular diseases.

### 3.6. Effects of Sitting on Frequency-Specific ISO

We examined sham-induced effects on the CWT-derived index of spectral amplitude (IA) and the index of spectral coherence (IC) of Δ[HbO] and Δ[oxCCO] by conducting one sample *t*-test (against baseline = 0) on all the sham-based results given in Figure 3 and Figure 4. The statistical analysis showed that ΔIA values of left and right Δ[HbO] were significantly different only in the myogenic band (*p* < 0.05) compared to their respective baselines. Specifically, we observed significant decreases in myogenic spectral amplitude bilaterally during the sham stimulation against their own baselines. This significant lessening in myogenic power could be attributed to the reduction in blood flow or circulation, which affects the smooth muscle activity of the blood vessel, perhaps as a result of sitting for 30 min or longer with limited head or body movement needed to minimize motion artifact [80].

### 3.7. Limitations and Future Works

Several limitations exist in this pilot study and need to be overcome for further confirmation of our findings as well as for clinical translations. First, the sample size of the study was very small (*n* = 12) and needs to be increased in future studies for the reproducibility and for better statistical power. Second, the measurement of bbNIRS was very motion sensitive, so it was difficult to acquire stable time series of Δ[HbO] and Δ[oxCCO] without much motion artifact over a long period of time (longer than 15 min). Further improvements on experimental protocol and setup as well as algorithm development to remove motion artifact are needed to allow a longer duration of high-quality recordings from the human brain. Third, since bbNIRS measurements interrogated the superficial layers (i.e., scalp and skull) of the human brain, they may have contributed to the measured Δ[HbO] and Δ[oxCCO] time series. Such contamination was not considered in this study and will be a future research topic to investigate.

## 4. Materials and Methods

### 4.1. Participants

Twelve healthy human subjects were recruited in the local community of the University of Texas at Arlington. They were screened by the same inclusion criteria as those in Wang et al. [32,81]. Each participant had two visits separated by 14 days to minimize post-tPBM residuals/effects. Each subject was subject to either tPBM or sham intervention; the sequence of tPBM and sham experiment was randomly assigned to each subject. Prior to each experiment, a written informed consent was signed by the participants. All participants were blind to tPBM or sham before they completed both experiments. The Institutional Review Board of the University of Texas at Arlington approved a total of 100 human participants for the study.

### 4.2. Experiment Protocol and Dual-Channel bbNIRS Setup

**Figure 5:**
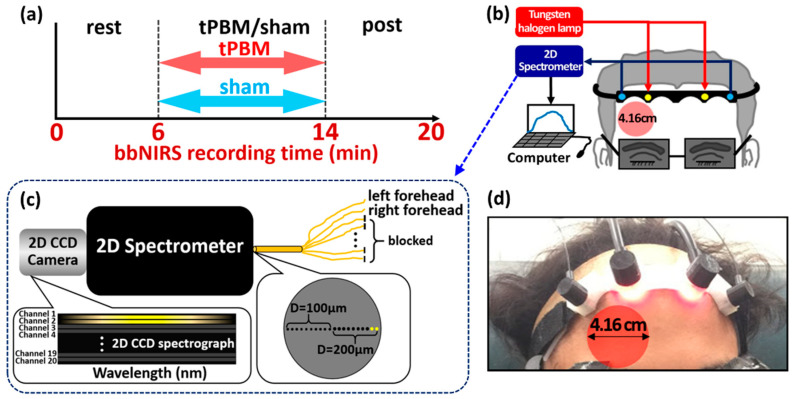
Experimental setup and protocols. (**a**) A schematic diagram showing the experimental protocol: the total experiment took 20 min, consisting of a 6-min resting/baseline period, an 8-min tPBM/sham period, and a 6-min post-tPBM/sham period. (**b**) A schematic diagram for the experimental setup, consisting of two pairs of bbNIRS placed on the right and left side of the forehead, which enabled concurrent readings before, during, and post tPBM/sham stimulations. (**c**) A schematic diagram to zoom in the 2D spectrometer/spectrograph and 2D CCD camera to achieve dual-channel bbNIRS. The large gray circle on the right bottom of the figure illustrates that all small detecting fibers (the small black circles) were arranged in a linear array at the entrance of the 2D spectrometer. Two diameters of fibers (100 and 200 µm) were implemented in the system (marked by D). In this study, however, we used only two channels of 200-μm fibers (small yellow circles) to collect optical data. (**d**) A photo of the actual probe holder setup on a human forehead: Four optical fiber bundles were steadily attached on the 3D-printed probe holder. The bbNIRS source-detector fiber separation was 3 cm; the laser (marked by the red circle) was delivered on the right forehead with an illumination diameter of 4.16 cm.

The experimental protocol for both tPBM and sham experiments is shown in Figure 5a. The total measurement/recording time was 20 min, including a 6-min resting period, an 8-min tPBM/sham period, and a 6-min post-tPBM/sham period. The participants were asked to comfortably sit on a 45-degree inclined sofa chair with their feet and legs flatly aligned on the leg-rest extension. Moreover, they were asked to close their eyes during the whole experiment procedure without falling asleep.

A dual-channel bbNIRS system was implemented in house and used to measure Δ[HbO] and Δ[oxCCO], respectively, from the bilateral prefrontal locations of the participants under resting state and post-tPBM/sham stimulations. Specifically, the experimental setup is schematically shown in Figure 5b. The system consisted of a broadband white light source (Model 3900e, Illumination Technologies, New York, NY, USA) and a two-dimensional CCD spectrograph (Teledyne Prinston Instrument, 3660 Quakerbridge Road, Trenton, NJ 08619, USA), allowing spectral recordings on a 1340 × 400 pixel CCD panel (Figure 5c). Two bbNIRS channels were placed bilaterally on the subject’s forehead, while tPBM/sham was delivered on the right prefrontal location with a 4.16-cm-diameter circular laser beam (see Figure 5d), similar to our previous work [34,36,82]. The ipsilateral bbNIRS channel enabled us to record the optical data adjacent to the tPBM site, while the other channel concurrently recorded data on the contralateral side. Each bbNIRS channel consisted of two fiber bundles, one for light delivery to the forehead and the other for light collection from brain tissue at 3 cm away from the source. The collected light was directed to the spectrograph/CCD camera. The diameter of the source fiber bundle was 3 mm, while the diameter of the detector fiber bundle was 200 µm. A probe holder was 3D printed with a flexible material. All the fiber bundles were perfectly fit into the probe holder, which was attached on the subject’s forehead comfortably by medical-grade bandage. Moreover, medical double-sided tapes were applied on the probe–skin interface to further secure the probe on the head and minimize motion artifacts.

### 4.3. tPBM at 1064 nm Delivered on Right Forehead

In this study, the device/hardware, dosage and stimulation locations of tPBM were the same as those published previously [32,34,82,83]. Briefly, active 1064-nm tPBM was continuously delivered for 8 min under a power density of 250 mW/cm^2^ on the right side of human foreheads between the eyebrow and hairline. The illumination size was circular with a diameter of 4.16 cm, leading to an illumination area of 13.6 cm^2^. In the case of sham stimulation, the laser power was set to be a minimum of 0.1 W while a black cap was also used to cover the laser aperture. The delivery of tPBM/sham was non-contact with the laser aperture positioned ~2 cm away from the forehead. Since the laser beam was well collimated, there was negligible difference in power density or irradiance between those at the laser aperture versus on the human forehead. A sensitive power meter (Model 843-R, Newport Corporation, Irvine, CA 92606, USA) was used to confirm the consistency of laser power at 0 and 2 cm away from the laser aperture. For both tPBM and sham experiments, all participants and operators wore a pair of protection goggles during the entire experimenters for eye protection.

### 4.4. Data Analysis

The raw data collected from the CCD camera were the time-dependent optical spectra with a temporal resolution of 4.04 Hz. In theory, the sampling rate of a spectrometer is proportional to the spectral range of light detected, an integration time window, and computer speed. In this study, the spectral range of interest was 780–900 nm, while the integration time used for the 2D spectrometer system was 200 ms. With a short delay (<200 ms for each sampling spectrum) caused by transferring and saving data in the computer, the actual sampling rate in our experiments turned out to be 4.04 Hz after being repeatedly verified with different lengths of data acquisition durations. In this study, the frequency range of interest was 0.005–0.2 Hz. Therefore, based on Nyquist Theorem, having a ~4 Hz of sampling frequency was more than adequate for frequency-dependent data analysis.

The selected spectral range for data processing was 780–900 nm, which was consistent with our previous work. Next, multi-variable linear regression introduced in Wang et al. [32] was performed to quantify Δ[HbO] and Δ[oxCCO] from each spectrum. After repeating the fitting processes at all the recorded time points, we obtained time series of Δ[HbO] and Δ[oxCCO] for the whole duration of the 20-min experiment. However, because of more frequent motion artifacts and drowsiness in subjects being observed toward the last two minutes of post stimulation, we kept 4 min (instead of 6 min) of post-tPBM/sham period in our data analysis.

In this study, our research interest was in baseline/pre-stimulation conditions versus post-stimulation conditions. Thus, data analyses on Δ[HbO] and Δ[oxCCO] time series were performed accordingly under rest (i.e., pre-tPBM/sham) and post-tPBM/sham conditions. We utilized two time-frequency analyses to investigate cerebral hemodynamic and metabolic oscillations before and after tPBM/sham. One was continuous wavelet transform (CWT), which quantifies a time-varying oscillation amplitude of Δ[HbO] and Δ[oxCCO] measured on each prefrontal side. The second analysis method was wavelet transform coherence (WTC) that affords quantification of wavelet coherence between each pair among Δ[HbO]_right_, Δ[HbO]_left_, Δ[oxCCO]_right_, and Δ[oxCCO]_left_. It was WTC calculations that defined and achieved the unilateral cerebral hemodynamic-metabolic coherence and bilateral functional connectivity of the human forehead at rest and post tPBM.

#### 4.4.1. CWT Analysis for the Human Prefrontal Cortex at Rest

**Figure 6:**
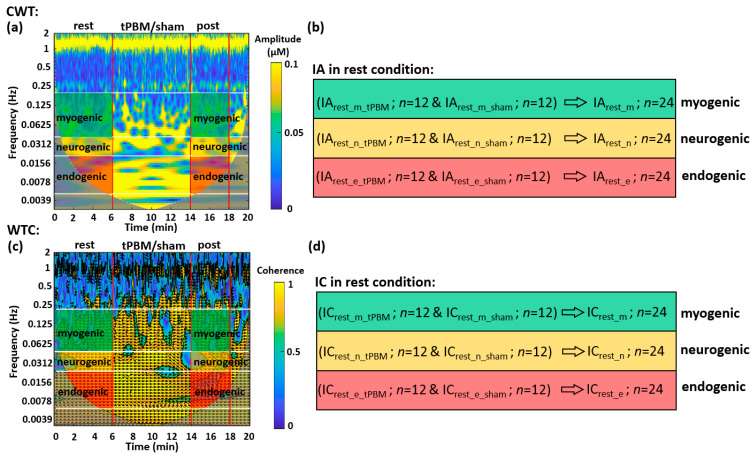
Example of CWT and WTC spectrograms presented in time-frequency maps and parameterized indexes for the data taken at rest. (**a**) It plots a CWT spectrogram generated from the right forehead of one subject. The vertical red lines segment the experimental time into three periods: a 6-min resting period, an 8-min tPBM period, and a 4-min post-tPBM/sham period. The white horizontal lines mark three frequency bands: endogenic (0.005–0.02 Hz; red-shaded), neurogenic (0.02–0.04 Hz; yellow-shaded), and myogenic (0.04–0.2 Hz; green-shaded) bands. The colormap indicates the time-frequency dependent amplitude of wavelet transform of the Δ[HbO] time series. Two gray-shaded areas indicate the cone of influence (COI) caused by the edge effects of wavelets and excluded for further data processing due to unreliability [84,85,86]. (**b**) It expresses the index of amplitude (IA) calculated by averaging CWT amplitude in the 2-dimensional, shaded areas given in (**a**) for respective ISO frequency bands. Subscripts of “rest_m_tPBM” and “rest_m_sham” mean under resting and sham conditions for tPBM experiment at myogenic frequency, respectively. The same notations apply to the other two situations. “*n* = 12” indicates the subject number for each of tPBM and sham experiments. For both baseline measurements, the total measurement number becomes 24. (**c**) It plots a WTC spectrogram generated from the coherence between two bilateral Δ[HbO] time series of one subject. Both red vertical and white horizontal lines serve the same purposes as those in (**a**). The arrows indicate the phase relationships between the two time series. The colormap marks time-frequency-dependent coherence amplitude between two bilateral Δ[HbO] time series. The gray-shaded COI areas were excluded for further data analysis. (**d**) It expresses the index of coherence (IC) for each ISO frequency band calculated by averaging shaded areas given in (**c**) for respective ISO frequency bands. While the red-, yellow-, and green-shaded areas in (**a**) and (**c**) are the regions of interest for calculations of IA and IC in three respective ISO frequency bands, they were separately quantified for the resting (or pre tPBM) and post-tPBM period.

CWT produces a spectrogram (i.e., time-frequency map), plotting the spectral amplitude as a function of time and frequency. Figure 6a is a CWT spectrogram of Δ[HbO] taken from the right forehead of one subject. The gray-shaded area is called ‘cone of inference’ (COI), within which the coherence values are not reliable [84,85,86] and thus were excluded for data analysis. To quantify an amplitude spectrum of the CWT spectrogram of Δ[HbO] time series at rest, we averaged CWT amplitude temporally (i.e., horizontally), making the 2-dimensional map into a 1-dimensional array. Note that the 6-min ‘rest’ segment before the tPBM or sham condition represented the resting state of the subjects without any intervention. Therefore, the amplitude spectra from both tPBM (*n* = 12) and sham conditions (*n* = 12) during ‘rest’ had no significant difference; thus, they were pooled (*n* = 24) for better statistical power. After group-averaging (*n* = 24) from 6-min baseline readings, we produced the amplitude spectra of the prefrontal cortex for all four metrics, Δ[HbO]_right_, Δ[HbO]_left_, Δ[oxCCO]_right_, and Δ[oxCCO]_left_, as shown in Figure 1a–d.

Furthermore, to parameterize the oscillation amplitude in each ISO frequency band, amplitude values from each temporal segment in the time-frequency map were two-dimensionally averaged to be one single value, as marked and described in Figure 6b. The time- and frequency-averaged CWT amplitude in each of the three ISO bands was defined as the Index of Amplitude (IA). Note that this process was performed within the shaded boxes for both the 6-min ‘rest’ and 4-min ‘post’ periods shown in Figure 6a; the latter was used when we investigated the post-tPBM/sham condition. The group-level IA values during ‘rest’ were pooled (*n* = 24; *n* = 12 from sham and *n* = 12 from tPBM condition) for a better statistical power and further combined as IA_rest_m_, IA_rest_n_, and IA_rest_e_, which denote IA values in myogenic, neurogenic, and endogenic bands, respectively, during rest condition (Figure 6b). For each subject, by repeating the above operations, IA values for Δ[HbO] and Δ[oxCCO] on both lateral sides of resting prefrontal cortex were obtained for each ISO band. Finally, group-averaged IA values were achieved for all three frequency bands on both prefrontal sides and plotted in Figure 1e,f for Δ[HbO] and Δ[oxCCO], respectively.

#### 4.4.2. WTC Analysis for the Human Prefrontal Cortex at Rest

WTC can reveal the time-locked behavior of two signals at different frequency components and during each period. In other words, performing WTC on two time series can provide both the time-dependent and frequency-dependent coherence and phase delay of the two signals. Specifically, in this study, WTC analysis enabled us to obtain coherence spectra between two bilateral Δ[HbO] time series and between two Δ[oxCCO] time series at rest, respectively, facilitated by MATLAB built-in function [86,87,88]. Similar to CWT, WTC produces spectrograms, displaying spectral coherence between two time series during a time period of the experiment. Specifically, Figure 6c shows an example of WTC coherence between two bilateral Δ[HbO] time series from one subject. The numeric values outside COI and within the black contours were considered reliable with 95% confidence [86] and thus included for further spectral coherence calculations. Moreover, the directions of the arrows denote the phase relationships between the two temporal signals at respective time points. In this study, we did not consider different phase angles (Figure 6c) and averaged all coherence values together for the next-step data analysis.

#### 4.4.3. Normalization of tPBM-Induced Changes in IA Values

In this study, we focused on post-tPBM/sham-induced effects on ISO amplitude and coherence, so comparisons of IA and IC between pre and post intervention were made by dividing and subtracting the respective 6-min baseline values, respectively. Specifically, we defined changes in IA, ΔIA, by normalization to the baseline IA, as follows:

(1) $${\Delta \mathrm{IA}}_{\left(i,j\right)}=\frac{IA_{post\left(i,j\right)}-IA_{rest\left(i,j\right)}}{IA_{rest\left(i,j\right)}}\times 100\%$$

where *i* denotes the frequency band that includes ‘e’ for endogenic, ‘n’ for neurogenic, and ‘m’ for myogenic band, *j* denotes the experiment condition for either tPBM or sham, and *IA_rest(i,j)_* and *IA_post(i,j)_* denote the IA values during the 6-min resting/pre-intervention and 4-min post-intervention period, respectively. The ratio, ΔIA_(*i,j)*_, is the normalized change in percentage in ISO amplitude at each of three frequency bands. This process was performed for all four metrics of Δ[HbO] and Δ[oxCCO] on both lateral sides of the forehead.

Statistical paired *t*-tests were performed to test the significant differences of each respective pair of IAs between two baseline values of *IA_rest(i,tPBM)_* and *IA_rest(i,sham)_*. This set of t-tests were performed for each of the four Δ[HbO] and Δ[oxCCO] metrics. No significant differences were observed in each respective pair, justifying the pooled measurement number of n = 24 for data analysis. Moreover, the statistical conclusion that the baseline IAs of tPBM and sham were identical confirmed that it was statistically appropriate to make baseline-normalized comparisons of respective ΔIA_(*i,j)*_ for Δ[HbO] and Δ[oxCCO] metrics between tPBM and sham conditions.

Next, another set of paired *t*-tests were performed to test the significance between the two intervention conditions, namely, ΔIA_(*i,tPBM)*_ versus ΔIA_(*i,sham)*_, where *i* can be any of the three frequency bands. This was performed for all four Δ[HbO] and Δ[oxCCO] metrics, producing the bar plots shown in Figure 3a,b.

#### 4.4.4. Baseline Subtraction of tPBM-Induced Changes in IC Values

For comparisons of IC values between pre and post intervention, we took the baseline-subtraction approach. Because some subjects had “zero” coherence baseline in the resting period, normalization with divisions would not work. Instead, subtraction was used to calibrate the ICs with respect to the resting baseline, as defined below:

(2) $${\Delta \mathrm{IC}}_{\left(i,j\right)}={\;\mathrm{IC}}_{post\left(i,j\right)}-{\mathrm{IC}}_{rest\left(i,j\right)}$$

where *i* denotes the frequency band that includes ‘e’ for endogenic, ‘n’ for neurogenic, and ‘m’ for myogenic band, *j* denotes the experiment condition for either tPBM or sham, and IC*_rest(i,j)_* and IC*_post(i,j)_* denote the IC values during the 6-min resting/pre-intervention and 4-min post-intervention period, respectively. The difference, ΔIC_(*i,j*)_, is the relative change in ISO coherence at each of three frequency bands. This process was performed for all respective coherence/coherences between bilateral Δ[HbO]s, bilateral Δ[oxCCO]s, as well as unilateral Δ[HbO] versus Δ[oxCCO] on both sides of the forehead. Finally, paired *t*-tests were conducted to examine significant differences in ΔIC values between tPBM and sham conditions for each frequency band, leading to the results shown in Figure 4a–d.

## 5. Conclusions

In this study, we introduced a novel methodology to non-invasively quantify bilateral functional connectivity and ipsilateral cerebral hemodynamic-metabolic coherence in the human prefrontal cortex by taking dual-channel bbNIRS measurements and performing CWT and WTC analyses on the measured bilateral Δ[HbO] and Δ[oxCCO] time series. The time-averaged WTC showed that both Δ[HbO] and Δ[oxCCO] held significant ISO consisting of endogenic, neurogenic, and myogenic frequencies. The quantified prefrontal connectivity and cerebral hemodynamic-metabolic coherence were defined and assessed by a wavelet index of coherence (i.e., IC values) ranging between 0 and 1 in each frequency band under resting state (or pre-) and post-tPBM/sham intervention. At rest, bilateral hemodynamic connectivity was very stable and consistent across all three bands with a group-level IC of 0.66, while bilateral ICs for metabolic connectivity were relatively weaker. Moreover, bilateral symmetry and resemblance of cerebral hemodynamic-metabolic coherence in the prefrontal cortex at rest were observed for all three bands. Moreover, the dual-channel bbNIRS measurements taken 4-min post tPBM/sham enabled us to achieve several key findings, namely, 8-min, right-forehead, 1064-nm tPBM (1) significantly enhanced the spectral amplitude of Δ[oxCCO] bilaterally, (2) promoted the bilateral neurogenic connectivity of CCO, and (3) made the main effect only on endothelial cells, leading to alteration of hemodynamic-metabolic coherence on each side of the prefrontal cortex. Overall, the quantifiable, absolute-valued metrics of both bilateral and ipsilateral (or unilateral) coherence indexes have the potential to become biomarkers or features for the detection of brain disorders in hemodynamic and/or metabolic activities by simple, short, resting-state recordings of dual-channel bbNIRS.

## Data Availability

The data presented in this study are available on request from the corresponding author since we have not setup a public archive platform for data sharing.
